# Aggressive papillary thyroid carcinoma in a child with type 2 congenital generalized lipodystrophy

**DOI:** 10.20945/2359-3997000000096

**Published:** 2019-02-01

**Authors:** Grayce Ellen da Cruz Paiva Lima, Virgínia Oliveira Fernandes, Ana Paula Dias Rangel Montenegro, Annelise Barreto de Carvalho, Lia Beatriz de Azevedo Sousa Karbage, Lindenberg Barbosa Aguiar, Mário Sérgio Rocha Macedo, Luis Alberto Albano Ferreira, Renan Magalhães Montenegro

**Affiliations:** 1 Universidade Federal do Ceará Universidade Federal do Ceará Hospital Universitário Walter Cantídio Grupo Brasileiro para Estudos de Lipodistrofias Herdadas e Adquiridas Fortaleza CE Brasil Grupo Brasileiro para Estudos de Lipodistrofias Herdadas e Adquiridas (BRAZLIPO), Hospital Universitário Walter Cantídio, Universidade Federal do Ceará (UFC), Fortaleza, CE, Brasil

## Abstract

Thyroid carcinoma (TC) is rare in children, particularly in those aged < 10 years. Several studies have demonstrated a correlation between neoplasms and hyperinsulinemia and insulin resistance, which are often associated with a higher risk for and/or aggressiveness of the neoplasm. Congenital generalized lipodystrophy (CGL) with autosomal recessive inheritance is a rare disease and is characterized by the lack of adipose tissue, severe insulin resistance, and early metabolic disturbances. Here, we reported a rare case of a type 2 CGL in a girl who presented with a papillary TC (PTC) at the age of 7 years. She had no family history of TC or previous exposure to ionizing radiation. She had a generalized lack of subcutaneous fat, including the palmar and plantar regions, muscle hypertrophy, intense acanthosis nigricans, hepatomegaly, hypertriglyceridemia, severe insulin resistance, and hypoleptinemia. A genetic analysis revealed a mutation in the *BSCL2* gene (p.Thr109Asnfs* 5). Ultrasound revealed a hypoechoic solid nodule measuring 1.8 × 1.0 × 1.0 cm, and fine needle aspiration biopsy suggested malignancy. Total thyroidectomy was performed, and a histopathological examination confirmed PTC with vascular invasion and parathyroid lymph node metastasis (pT3N1Mx stage). This is the first report to describe a case of differentiated TC in a child with CGL. Severe insulin resistance that is generally observed in patients with CGL early in life, especially in those with type 2 CGL, may be associated with this uncommon presentation of aggressive PTC during childhood.

## INTRODUCTION

Thyroid carcinoma (TC) is rare in children, particularly those aged < 10 years, and accounts for 1.5%–3% of all childhood cancers in North America and Europe; however, its incidence has been increasing by 1.1% each year (1). In Brazil, according to the National Cancer Institute database, the incidence of TC may be 2% of all pediatric cancers (2). The behavior of TC in children aged < 10 years differs from that in adults, because in the pediatric range there is usually at least one risk factor for papillary TC (PTC), mainly ionizing radiation exposure and family history (3).

Several studies have revealed the correlation between neoplasms and hyperinsulinemia and insulin resistance (IR), which are frequently associated with a higher risk for and/or aggressiveness of the neoplasm (4–6). However, many of those studies included subjects who were obese, which characterizes many confounding variables (6–8), and data in conditions of severe IR are lacking.

CGL is a rare disease with autosomal recessive inheritance characterized by a lack of adipose tissue, hypoleptinemia, severe IR, and early metabolic disturbances such as dyslipidemia and diabetes mellitus (9,10).

Here, we report a rare case of type 2 CGL in a girl who presented with PTC at the age of 7 years and who had no family history of TC or previous exposure to ionizing radiation.

## CASE REPORT

A 9-year-old girl with a clinical and molecular diagnosis of CGL was being followed at our center, (BRAZLIPO Program, Endocrine and Diabetes Unit, University Hospital, Federal University of Ceará, Brazil). Based on the generalized absence of adipose tissue, she was clinically diagnosed as having CGL in her first year of life.

**Figure 1:**
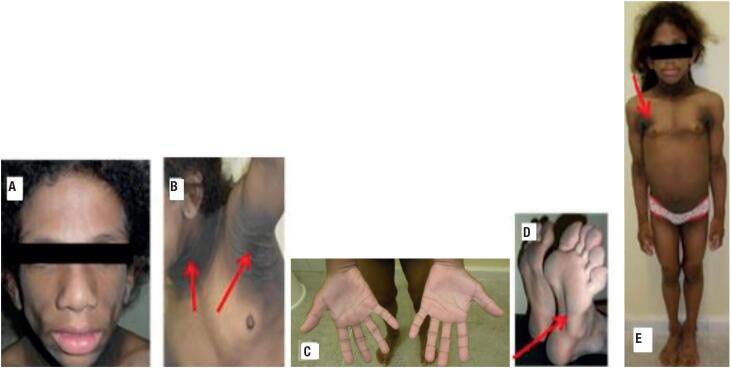
Clinical characteristics of a 7-year-old girl with a diagnosis of congenital generalized lipodystrophy. (**A**) Absence of adipose tissue in the face. (**B**) Severe acanthosis nigricans. (**C** and **D**) Absence of adipose tissue in the palmar and plantar regions, respectively. (**E**) Global absence of adipose tissue and muscle pseudohypertrophy.

**Figure 2:**
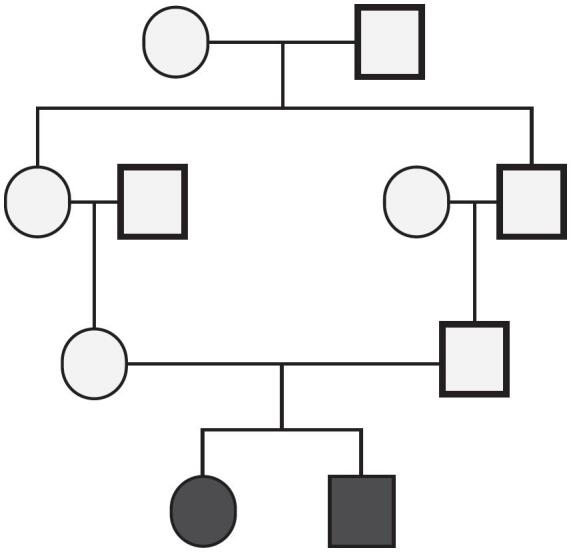
The family pedigree of the patient showing two siblings affected by congenital generalized lipodystrophy.

A physical examination at 7 years of age revealed a height of 143 cm, a weight of 42.1 kg, and a body mass index (BMI) of 20.5 kg/m^2^. She had a generalized lack of subcutaneous fat, including in the palmar and plantar regions, muscle hypertrophy, intense acanthosis nigricans, and hepatomegaly (Figure 1). The patient also had a history of consanguinity. Her parents are first-degree cousins, and her brother has the same clinical diagnosis of CGL (Figure 2).

In her first year of life, hypertriglyceridemia (442 mg/dL) was detected. During follow-up, her clinical and laboratory work-up revealed IR (Table 1). At 7 years of age, fasting hyperinsulinemia, severe IR, abnormal glucose tolerance, and hypoleptinemia were also observed. At this time, a genetic analysis revealed a *BSCL2* gene mutation (p.Thr109Asnfs* 5), characterizing type 2 CGL.

**Table 1:** Clinical and laboratory data of a patient with CGL during follow-up

	First evaluation	Second evaluation	Third evaluation
Age (years)	2.16	4.75	7.58
Weight	15.5	27.7	42.1
Height	96	122.5	143
BMI (kg/m^2^)	16.6	18.4	20.5
Glucose (mg/dL)	72	80	73
A1c (%)	–	6,3	6,1
Insulin (µU/mL)	20.6	41.02	102
HOMA-IR	3.66	8.09	8,3
TC (mg/dL)	185	143	161
HDL (mg/dL)	22	23	28
Triglycerides (mg/dL)	284	324	117
TSH (µUI/mL)	2.09	1.2	2.3
Leptin (ng/mL)	–	1.0	–

BMI: body mass index; HOMA: homeostasis model assessment-insulin resistance; TC: total cholesterol; HDLc: high-density lipoprotein cholesterol; TG: triglyceride; TSH: thyroid-stimulating hormone.

At 7 years of age, thyroid gland ultrasound revealed a hypoechoic solid nodule measuring 1.8 × 1.0 × 1.0 cm in size, with partially defined margins and multiple foci of microcalcification located in the upper third of the right lobe. According to the clinical history, the patient had no family history of TC or previous exposure to ionizing radiation.

**Figure 3:**
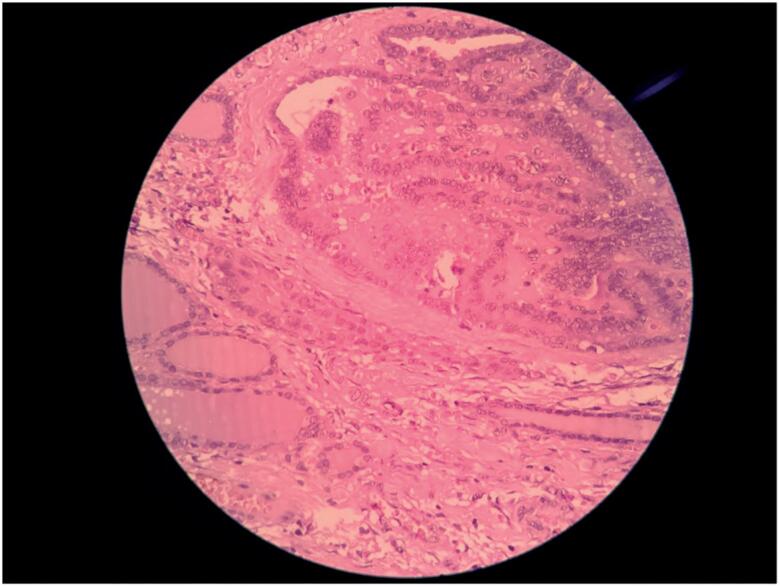
Histopathological examination showing normal thyroid tissue below and papillary thyroid neoplasia at the top of the image.

Results of a cytological evaluation of the nodule, obtained through fine-needle aspiration biopsy, suggested malignancy (Bethesda V classification). Thus, total thyroidectomy was performed. Results of a histopathological examination confirmed a classical variant of PTC, with the tumor measuring 1.2 cm (unifocal) and having vascular invasion. Perineural infiltration was not performed. Extrathyroid extension and metastasis were observed in a parathyroid lymph node (pT3N1Mx stage) (Figure 3). The patient also underwent radioiodine therapy (100 mCi), followed by suppressive levothyroxine treatment (3 mcg/kg/day), and no signs of residual disease.

## RESULTS AND DISCUSSION

This is the first study to report about a differentiated TC (DTC) in a child with CGL. Although the patient did not present with other risk factors for TC, i.e., previous radiation exposure or a family history of TC, she had severe CGL-associated IR since early childhood. Furthermore, she had an aggressive presentation of PTC (pT3N1Mx stage).

CGL is a rare congenital disorder that is characterized by total or near-total lack of body fat, low leptin levels, ectopic fat deposits, and severe metabolic disorders such as hypertriglyceridemia and IR (10). Four subtypes of CGL have been described. Subtypes 1 and 2 are responsible for 95% of described cases and occur because of mutations in the *AGPAT2* and *BSL2* genes, respectively, which express proteins that are critical in triglyceride and phospholipid biosynthesis, lipid droplet formation, and adipocyte differentiation (11). Low leptin and adiponectin levels, increased free fatty acids, and triglyceride depositions in the liver and skeletal muscles, which are associated with severe IR, are frequently noted (12–15).

CGL because of *BSCL2* mutation is the most severe form of CGL, with a pronounced lack of adipose tissue (16). It affects both the metabolic active and mechanical adipose tissues and usually results in earlier and more severe metabolic abnormalities. Mental retardation and cardiomyopathy have also been described (16). Our patient presented with clinical and biochemical phenotypic characteristics of CGL.

She had hypertriglyceridemia and intense acanthosis nigricans since her first year of life. The first described case of the association between DTC and IR was in a 13.5-year-old obese girl with acanthosis nigricans (17). Diabetes and IR are considered risk factors for DTC (18). Epidemiological studies have suggested the association, although in such studies only obese subjects were evaluated, possibly leading to the presence of a positive confounder (7,19,20). A previous cross-sectional study assessed the correlation between IR and thyroid, independent of body weight, in participants grouped according to their BMI and homeostasis model assessment-IR (HOMA-IR) and concluded that individuals with higher IR had a higher number of nodules and greater thyroid volume, independent of BMI (8).

Our patient had hyperinsulinemia since the 7 years of age. Insulin acts as a growth factor that stimulates cell proliferation. Some studies have described that insulin receptors (IRs) are overexposed in most DTC cases (21,22). In addition, insulin reduces insulin-like growth factor-binding protein levels, leading to increased levels of insulin-like growth factor (IGF), subsequently resulting in cell multiplication and apoptosis reduction. Insulin also increases the risk of mutations and cancer development (22).

IGF-1 plays a role in various human malignancies and possibly in DTC (21,22). IGF-1 is important for thyroid growth and development, as thyroid-stimulating hormone (TSH). In addition, IGF-receptors are overexpressed in DTC (21), which amplifies the anabolic effect on thyrocytes (23).

Some studies have described the presence of high leptin levels in patients with DTC (24). Cheng and cols. demonstrated that leptin and leptin receptors are overexpressed in patients with PTC, which correlates with increased tumor aggressiveness (7). In contrast, our patient had very low leptin levels, which correlated to the absence of metabolic active adipose tissues.

Although our patient had no clinical diagnosis of diabetes, she had altered glycated hemoglobin levels. Most studies have demonstrated the correlation between diabetes and DTC. In their case-control study, Bae and cols. observed that Korean women in the upper quarters of HOMA-IR had an odds ratio of 4.07 (95% confidence interval [CI] 2.81–5.89, p < 0.001) for DTC. These data were adjusted for age, BMI, history of smoking, hypertension, thyroid disease, other cancers, cholesterol levels and TSH (25).

In the meta-analysis conducted by Yeo and cols., women with pre-existing DM had an increased risk for TC (relative risk = 1.38, 95% CI 1.13–1.67) (26). A similar conclusion was observed in another review that revealed that diabetes had a possible positive association with risk for TC (27). Conversely, in a systematic review, Kitahara and cols. concluded that neither physical inactivity nor diabetes history was associated with an increased risk for TC (28). These results are controversial, and evidence is not sufficient to associate RI or diabetes with TC (25,29,30).

However, it is unlikely that the association reported here is a random occurrence. The occurrence of DTC in children aged < 10 years is rare and is usually associated with the presence of identifiable risk factors such as a family history and exposure to ionizing radiation during childhood; however, this was not observed in our patient. Our patient had IR since her first year of life, which was related to the most severe subtype of CGL. Our case findings reinforce the hypothesis that IR is a trigger for CGL, independent of BMI or leptin levels.

In conclusion, this is the first report to describe DTC in a child with CGL. The severe IR usually observed in this disorder early in life, especially type 2 of CGL, may be associated with the uncommon presentation of aggressive PTC during childhood. Future prospective studies are needed to better define the association between TC and severe IR and demonstrate the possible benefit of thyroid evaluation in patients with CGL.

## References

[1] Greenlee RT, Hill-Harmon MB, Murray T, Thun M. Cancer statistics, 2001. CA Cancer J Clin. 2001;51(1):15-36. 10.3322/canjclin.51.1.15 11577478

[2] Ministry of Health, National Cancer Institute (INCA); Brazilian Society of Pediatric Oncology (SOBOPE). Childhood and Adolescent Cancer in Brazil: Data from Mortality and population based-registries. Rio de Janeiro: INCA; 2009 [access by 23 mar 2018]. Available at: http://www1.inca.gov.br/tumores_infantis/pdf/English_Version_tumores_Infantis06102009.pdf.

[3] Rivkees SA, Mazzaferri EL, Verburg FA, Reiners C, Luster M, Breuer CK, et al. The treatment of differentiated thyroid cancer in children: emphasis on surgical approach and radioactive iodine therapy. Endocr Rev. 2011;32(6):798-826. 10.1210/er.2011-0011 PMC3591676 21880704

[4] Arcidiacono B, Iiritano S, Nocera A, Possidente K, Nevolo MT, Ventura V, et al. Insulin resistance and cancer risk: an overview of the pathogenetic mechanisms. Exp Diabetes Res. 2012;2012:789174. 10.1155/2012/789174 PMC3372318 22701472

[5] Tsugane S, Inoue M. Insulin resistance and cancer: epidemiological evidence. Cancer Sci. 2010;101(5):1073-9. 10.1111/j.1349-7006.2010.01521.x PMC11159937 20345478

[6] Rezzónico JN, Rezzónico M, Pusiol E, Pitoia F, Niepomniszcze H. Increased prevalence of insulin resistance in patients with differentiated thyroid carcinoma. Metab Syndr Relat Disord. 2009;7(4):375-80. 10.1089/met.2008.0062 19320560

[7] Cheng SP, Chi CW, Tzen CY, Yang TL, Lee JJ, Liu TP, et al. Clinicopathologic significance of leptin and leptin receptor expressions in papillary thyroid carcinoma. Surgery. 2010;147(6):847-53. 10.1016/j.surg.2009.11.004 20045163

[8] Rezzonico J, Rezzonico M, Pusiol E, Pitoia F, Niepomniszcze H. Introducing the thyroid gland as another victim of the insulin resistance syndrome. Thyroid. 2008;18(4):461-4. 10.1089/thy.2007.0223 18346005

[9] Patni N, Garg A. Congenital generalized lipodystrophies – new insights into metabolic dysfunction. Nat Rev Endocrinol. 2015;11(9):522-34. 10.1038/nrendo.2015.123 PMC7605893 26239609

[10] Van Maldergem L, Magré J, Khallouf TE, Gedde-Dahl T Jr, Delépine M, Trygstad O, et al. Genotype-phenotype relationships in Berardinelli-Seip congenital lipodystrophy. J Med Genet. 2002;39(10):722-33. 10.1136/jmg.39.10.722 PMC1734991 12362029

[11] Szymanski KM, Binns D, Bartz R, Grishin NV, Li WP, Agarwal AK, et al. The lipodystrophy protein seipin is found at endoplasmic reticulum lipid droplet junctions and is important for droplet morphology. Proc Natl Acad Sci U S A. 2007;104(52):20890-5. 10.1073/pnas.0704154104 PMC2409237 18093937

[12] Pardini VC, Victória IM, Rocha SM, Andrade DG, Rocha AM, Pieroni FB, et al. Leptin levels, beta-cell function, and insulin sensitivity in families with congenital and acquired generalized lipoatropic diabetes. J Clin Endocrinol Metab. 1998;83(2):503-8. 10.1210/jcem.83.2.4567 9467565

[13] Storz P, Döppler H, Wernig A, Pfizenmaier K, Müller G. Cross‐talk mechanisms in the development of insulin resistance of skeletal muscle cells. Eur J Biochem. 1999;266(1):17-25. 10.1046/j.1432-1327.1999.00809.x 10542046

[14] Carvalho MHC, Colaço AL, Fortes ZB. Citocinas, disfunção endotelial e resistência à insulina. Arq Bras Endocrinol Metabol. 2006;50(2):304-12. 10.1590/s0004-27302006000200016 16767296

[15] Garg A, Chandalia M, Vuitch F. Severe islet amyloidosis in congenital generalized lipodystrophy. Diabetes Care. 1996;19(1):28-31. 10.2337/diacare.19.1.28 8720529

[16] Simha V, Garg A. Phenotypic heterogeneity in body fat distribution in patients with congenital generalized lipodystrophy caused by mutations in the AGPAT2 or seipin genes. J Clin Endocrinol Metab. 2003;88(11):5433-7. 10.1210/jc.2003-030835 14602785

[17] Vassilopoulou-Sellin R, Cangir A, Samaan NA. Acanthosis nigricans and severe insulin resistance in an adolescent girl with thyroid cancer: clinical response to antineoplastic Am J Clin Oncol. 1992;15(3):273-6. 10.1097/00000421-199206000-00019 1590285

[18] Pappa T, Alevizaki M. Obesity and thyroid cancer: a clinical update. Thyroid. 2014;24(2):190-9. 10.1089/thy.2013.0232 23879222

[19] Agate L, Lorusso L, Elisei R. New and old knowledge on differentiated thyroid cancer epidemiology and risk factors. J Endocrinol Invest. 2012;35(6 Suppl):3-9. 23014067

[20] Davies L, Welch HG. Increasing incidence of thyroid cancer in the United States, 1973-2002. JAMA. 2006;295(18):2164-7. 10.1001/jama.295.18.2164 16684987

[21] Vella V, Sciacca L, Pandini G, Mineo R, Squatrito S, Vigneri R, et al. The IGF system in thyroid cancer: new concepts. Mol Pathol. 2001;54(3):121-4. 10.1136/mp.54.3.121 PMC1187048 11376121

[22] Belfiore A, Pandini G, Vella V, Squatrito S, Vigneri R. Insulin/IGF-I hybrid receptors play a major role in IGF-I signaling in thyroid cancer. Biochimie. 1999;81(4):403-7. 10.1016/s0300-9084(99)80088-1 10401676

[23] Eggo MC, Bachrach LK, Burrow GN. Interaction of TSH, insulin and insulin-like growth factors in regulating thyroid growth and function. Growth Factors. 1990;2(2-3):99-109. 10.3109/08977199009071497 2160262

[24] Hedayati M, Yaghmaei P, Pooyamanesh Z, Zarif Yeganeh M, Hoghooghi Rad L. Leptin: a correlated Peptide to papillary thyroid carcinoma? J Thyroid Res. 2011;2011:832163. 10.4061/2011/832163 PMC3189603 22007338

[25] Bae MJ, Kim SS, Kim WJ, Yi YS, Jeon YK, Kim BH, et al. High prevalence of papillary thyroid cancer in Korean women with insulin resistance. Head Neck. 2016;38(1):66-71. 10.1002/hed.23848 25196854

[26] Yeo Y, Ma SH, Hwang Y, Horn-Ross PL, Hsing A, Lee KE, et al. Diabetes mellitus and risk of thyroid cancer: a meta-analysis. PLoS One. 2014;9(6):e98135. 10.1371/journal.pone.0098135 PMC4057085 24927125

[27] Schmid D, Behrens G, Jochem C, Keimling M, Leitzmann M. Physical activity, diabetes, and risk of thyroid cancer: a systematic review and meta-analysis. Eur J Epidemiol. 2013;28(12):945-58. 10.1007/s10654-013-9865-0 24243033

[28] Kitahara CM, Platz EA, Beane Freeman LE, Black A, Hsing AW, Linet MS, et al. Physical activity, diabetes, and thyroid cancer risk: a pooled analysis of five prospective studies. Cancer Causes Control. 2012;23(3):463-471. 10.1007/s10552-012-9896-y PMC3586378 22294499

[29] Aschebrook-Kilfoy B, Sabra MM, Brenner A, Moore SC, Ron E, Schatzkin A, et al. Diabetes and thyroid cancer risk in the National Institutes of Health-AARP Diet and Health Study. Thyroid. 2011;21(9):957-63. 10.1089/thy.2010.0396 PMC3162644 21767143

[30] Luo J, Phillips L, Liu S, Wactawski-Wende J, Margolis KL. Diabetes, diabetes treatment, and risk of thyroid cancer. J Clin Endocrinol Metab. 2016;101(3):1243-8. 10.1210/jc.2015-3901 PMC4803153 26760177

